# Supplementary material for: Mechanisms and Drivers for the Establishment of Life Cycle Complexity in Myxozoan Parasites
Source: Biology (Basel). 2020 Jan 1;9(1):10. doi: 10.3390/biology9010010 (PMC7168919; doi:10.3390/biology9010010)
Supplement: Supplementary file 1 [file biology-09-00010-s001.zip › Supplementary references of Table S1.docx]

References

1. Thélohan, P. Observations sur les Myxosporidies et essai de classification de ces organismes. *Bull. Soc. Philom.* **1892**, *4*, 165–178.
2. Jameson, A.P. Notes on Californian Myxosporidia. *J. Parasitol.* **1931**, *18*, 59–68.
3. Kudo, R.R. A taxonomic consideration of Myxosporidia. *Trans. Am. Microsc. Soc.* **1933**, *52*, 195–216.
4. Molina, J.M.; Cazorla, A.L. Biology of *Myliobatis goodei* (Springer, 1939), a widely distributed eagle ray, caught in northern Patagonia. *J. Sea Res.* **2015**, *95*, 106–114, doi:10.1016/j.seares.2014.09.006.
5. Rocha, S.; Casal, G.; Al-Quraishy, S.; Azevedo, C. Morphological and molecular characterization of a new myxozoan species (Myxosporea) infecting the gall bladder of *Raja clavata* (Chondrichthyes), from the Portuguese Atlantic Coast. *J. Parasitol.* **2013**, *99*, 307–318, doi: 10.1645/GE-3150.1.
6. Kovaljova, A.A. Myxosporidia of the genus *Chloromyxum* (Cnidospora, Myxosporea) of cartilaginous fish from the Atlantic coast of Africa. *Parasitologiya* **1988**, *22*, 384–388.
7. Awruch, C.A.; Frusher, S.D.; Stevens, J.D.; Barnett, A. Movement patterns of the draughtboard shark *Cephaloscyllium laticeps* (Scyliorhinidae) determined by passive tracking and conventional tagging. *J. Fish Biol*. **2012**, *80*, 1417–1435, doi:10.1111/j.1095-8649.2012.03249.x.
8. Stelbrink, B.; von Rintelen, T.; Cliff, G.; Kriwet, J. Molecular systematics and global phylogeography of angel sharks (genus *Squatina*). *Mol. Phylogenet. Evol.* **2010**, *54*, 395–404, doi:10.1016/j.ympev.2009.07.029.
9. Mingazzini, P. Sullo sviluppo dei Myxosporidi. *Boll. Soc. Nat. Napoli* **1890**, *4*, 160–164.
10. Fiala, I.; Dyková, I. The phylogeny of marine and freshwater species of the genus *Chloromyxum* Mingazzini, 1890 (Myxosporea: Bivalvulida) based on small subunit ribosomal RNA gene sequences. *Folia Parasitol*. **2004**, *51*, 211–214.
11. Rocha, S.; Casal, G.; Al-Quraishy, S.; Azevedo, C. Morphological and ultrastructural redescription of *Chloromyxum leydigi* Mingazzini, 1890 (Myxozoa: Myxosporea), type species of the genus, infecting the gall bladder of the marine cartilaginous fish *Torpedo marmorata* Risso (Chondrichthyes: Torpedinidae), from the Portuguese Atlantic coast. *Folia Parasitol.* **2014**, *61*, 1–10, doi:10.14411/fp.2014.006.
12. Veríssimo, A.; McDowell, J.R.; Graves, J.E. Population structure of a deep-water squaloid shark, the Portuguese dogfish (*Centroscymnus coelolepis*). *ICES J. Mar. Sci.* **2011**, *68*, 555–563, doi:10.1093/icesjms/fsr003.
13. Kudo, R.R. *Studies on Myxosporidia: A Synopsis of Genera and Species of Myxosporidia*; University of Illinois: Urbana, IL, USA, 1920.
14. Dunkerly, J.S. Fish Myxosporidia from Plymouth. *Parasitology* **1910**, *12*, 328–333.
15. Vandeperre, F.; Aires-da-Silva, A.; Fontes, J.; Santos, M.; Santos, R.S.; Afonso, P. Movements of blue sharks (*Prionace glauca*) across their life history. *PLoS ONE* **2014**, *9*, e103538, doi:10.1371/journal.pone.0103538.
16. Kuznetsova, I.G. Myxosporidians of Chondrostei from the Patagonian shelf. *Parazitologiya* **1977**, *11*, 74–77.
17. Serena, F.; Papacostantinou, C.; Relini, G.; Gil De Sola, L.; Bertrand, J. Distribution and Abundance of Spiny Dogfish in the Mediterranean Sea based on the Mediterranean International Trawl Surveys Program. In *Biology and Management of Dogfish Sharks*; Gallucci, V.F., McFarlane, G.A., Bargmann, G.G., Eds.; American Fisheries Society: Bethesda, MD, USA, 2009; pp. 139–149.
18. Awerinzew, S. Ergebnisse der Intersuchungen über parasitische Protozoen der tropischen Region Afrikas. *Zool. Anz.* **1913**, *42*, 151–156.
19. Moser, M.; Kent, M.L.; Dennis, D. Gall-bladder Myxosporea in coral-reef fishes from Heron Island, Australia. *Aust. J. Zool.* **1989**, *37*, 1–13, doi:10.1071/ZO9890001.
20. Woolcock, V. *Chloromyxum pristiophori*, a new species of Myxosporidia parasitic in the gall-bladder of *Pristiophorus cirratus* (saw-shark). *Parasitology* **1936**, *28*, 72–78.
21. Azevedo, C.; Casal, G.; Garcia, P.; Matos, P.; Teles-Grilo, L.; Matos, E. Ultrastructural and phylogenetic data of *Chloromyxum riorajum* sp. nov. (Myxozoa), a parasite of the stingray *Rioraja agassizii* in Southern Brazil. *Dis. Aquat. Organ.* **2009**, *85*, 41–51, doi:10.3354/dao02067.
22. Branch, G.M.; Branch, M.L.; Griffiths, C.L.; Beckley, L.E. *Two Oceans: A Guide to the Marine Life of Southern Africa;* Struik Nature: Cape Town, South Africa, 2010.
23. Michael, S.W. *Reef Sharks and Rays of the World. A Guide to Their Identification, Behavior, and Ecology.* Sea Challengers: Monterey, CA, USA, 1993; pp. 1–107.
24. Noble, E.R. A new Myxosporidian (Protozoan) parasite from *Scyliorhinus torazame*. *Trans. Am. Microsc. Soc.* **1948**, *67*, 254–256, doi:10.2307/3223188.
25. Gioia, I.; Cordeiro, N.S. Brazilian Myxosporidians checklist (Myxozoa). *Acta Parasitol.* **1996**, *35*, 137–149.
26. Love, M.S.; Moser, M. *A Checklist of Parasites of California, Oregon, and Washington Marine and Estuarine Fishes*; NOAA Tech. Rep. NMFS SSRF-777; U.S. Dept. of Commerce, National Oceanic and Atmospheric Administration, National Marine Fisheries Service, Washington, DC, USA, 1983; pp. 1–576.
27. Gleeson, R.J.; Bennett, M.B.; Adlard, R.D. First taxonomic description of multivalvulidan myxosporean parasites from elasmobranchs: *Kudoa hemiscylli* n. sp. and *Kudoa carcharhini* n. sp. (Myxosporea: Multivalvulidae). *Parasitology* **2010**, *137*, 1885–1898, doi:10.1017/S0031182010000855.
28. Escalle, L.; Speed, C.W.; Meekan, M.G.; White, W.T.; Babcock, R.C.; Pillans, R.D.; Huveneers, C. Restricted movements and mangrove dependency of the nervous shark *Carcharhinus cautus* in nearshore coastal waters. *J. Fish Biol.* **2015**, *87*, 323–341, doi:10.1111/jfb.12724.
