# Supplementary material for: Mechanisms and Drivers for the Establishment of Life Cycle Complexity in Myxozoan Parasites
Source: Biology (Basel). 2020 Jan 1;9(1):10. doi: 10.3390/biology9010010 (PMC7168919; doi:10.3390/biology9010010)
Supplement: Supplementary file 1 [file biology-09-00010-s001.zip › Supplementary tablesS2-S4 figuresS1-S4.docx]

**Table S2:** GenBank accession numbers of myxozoan SSU rDNA sequences used in phylogenetic analysis (species from Chondrichthyes in bold)

| **Myxozoan species** | **Acces. Nr.** | **Myxozoan species** | **Acces. Nr.** |
| --- | --- | --- | --- |
| *Auerbachia pulchra* | DQ377703 | *Kudoa quadricornis* | FJ792720 |
| *Bipteria lusitanica* | MF538777 | *Kudoa scomberomori* | AY302737 |
| ***Bipteria vetusta*** | **KM267557** | *Kudoa shiomitsui* | LC128646 |
| *Buddenbrockia plumatellae* | KF731682 | *Kudoa thunni* | LC200502 |
| *Ceratomyxa arcuata* | KM273023 | *Kudoa thyrsites* | AY152747 |
| *Ceratomyxa auerbachi* | EU616734 | *Sphaerospora fugu* | AB195805 |
| *Ceratomyxa brasiliensis* | KU978813 | *Myxidium anatidum* | EF602629 |
| *Ceratomyxa brayi* | EU729697 | *Myxidium baueri* | JX467674 |
| *Ceratomyxa buri* | AB530264 | *Myxidium ceccarellii* | KJ499821 |
| ***Ceratomyxa carcharhini*** | **JF911816** | *Myxidium coryphaenoideum* | DQ377697 |
| *Ceratomyxa informis* | KM273022 | *Myxidium hardella* | AY688957 |
| *Ceratomyxa leatherjacketi* | KM273028 | *Myxidium laticurvum* | JN033230 |
| ***Ceratomyxa melanopteri*** | **JF911817** | *Myxidium lieberkuehni* | X76639 |
| *Ceratomyxa moseri* | EU440360 | *Myxidium scripta* | DQ851568 |
| ***Ceratomyxa negaprioni*** | **JF911818** | *Myxidium streisingeri* | KM001688 |
| *Ceratomyxa sparusaurati* | AF411471 | *Myxobilatus gasterostei* | EU861210 |
| ***Ceratomyxa* sp. ex *Scyliorhinus canicula*** | **DQ377698** | *Myxobolus catlae* | KM029967 |
| ***Ceratomyxa* sp. 1 ex *Myliobatis goodei*** | **MK937839** | *Myxobolus cerebralis* | EF370479 |
| ***Ceratomyxa* sp. 2 ex *Myliobatis goodei*** | **MK937835** | *Myxobolus honghuensis* | KR049222 |
| ***Ceratomyxa* sp. 1 ex *Sphyrna tiburo*** | **MK937837** | *Myxobolus neurotropus* | DQ846661 |
| ***Ceratomyxa* sp. 2 ex *Sphyrna tiburo*** | **MK937838** | *Myxodavisia bulani* | KM273030 |
| ***Ceratomyxa* sp. ex *Myliobatis ridens*** | **MK937840** | *Ortholinea auratae* | KR025869 |
| ***Ceratomyxa* sp. ex *Rostroraja eglanteria*** | **MK937836** | *Ortholinea mullusi* | MF539825 |
| *Ceratomyxa synaphobranchi* | KM273026 | *Ortholinea orientalis* | HM770873 |
| *Ceratomyxa tunisiensis* | KT013098 | *Ortholinea* sp. ex *Dicentrarchus labrax* | KU363830 |
| *Ceratomyxa vermiformis* | KX278420 | ***Ortholinea* sp. ex *Rhizoprionoton terraenovae*** | **MK937851** |
| *Ceratonova gasterostea* | KF751186 | *Palliatus indecorus* | DQ377712 |
| *Ceratonova shasta* | GQ358729 | *Parvicapsula anisocaudata* | KY341924 |
| *Coccomyxa gobiodoni* | HM037791 | *Parvicapsula bicornis* | EF429097 |
| *Cystodiscus melleni* | DQ003031 | *Parvicapsula curvatura* | MF161398 |
| *Ellipsomyxa adlardi* | JX443488 | *Parvicapsula kabatai* | DQ515821 |
| *Enteromyxum leei* | MF161396 | *Parvicapsula limandae* | EF429096 |
| *Gadimyxa arctica* | EU163426 | *Parvicapsula minibicornis* | HQ624977 |
| *Gadimyxa atlantica* | EU163412 | *Parvicapsula petuniae* | KF874230 |
| *Gastromyxum rafii* | KT002406 | *Parvicapsula pseudobranchicola* | AY308481 |
| *Henneguya psorospermica* | EU732602 | *Parvicapsula spinachiae* | EF431928 |
| *Henneguya salminicola* | AF031411 | ***Parvicapsula* sp. ex *Sphyrna tiburo*** | **MK937852** |
| *Hoferellus* sp. *alosae* | KU301052 | *Sinuolinea dimorpha* | JX460905 |
| ***Chloromyxum alantoraji*** | **MG652633** | *Sinuolinea phyllopteryxa* | DQ645952 |
| ***Chloromyxum clavatum*** | **JQ793641** | *Soricimyxum fegati* | KU248478 |
| *Chloromyxum fluviatile* | GU471264 | *Sphaeromyxa hellandi* | DQ377701 |
| ***Chloromyxum hemiscylli*** | **JN130374** | *Sphaeromyxa lycodi* | KC524734 |
| ***Chloromyxum kuhlii*** | **JN130375** | *Sphaerospora angulata* | JQ801526 |
| ***Chloromyxum lesteri*** | **JN130377** | *Sphaerospora dicentrarchi* | KT970638 |
| ***Chloromyxum leydigi*** | **AY604199** | *Sphaerospora dykovae* | JQ801532 |
| ***Chloromyxum leydigi*** | **DQ377710** | *Sphaerospora elegans* | JX286618 |
| ***Chloromyxum mingazzinii*** | **JN130379** | *Sphaerospora formosa* | FJ790307 |
| ***Chloromyxum myliobati*** | **JN130380** | *Sphaerospora motemarini* | KC526873 |
| ***Chloromyxum riorajum*** | **FJ624481** | *Sphaerospora ohlmacheri* | JX286619 |
| ***Chloromyxum* sp. ex *Squalus acanthias*** | **JN130384** | *Sphaerospora olsoni* | KJ526213 |
| ***Chloromyxum* sp. ex *Sphyrna tiburo*** | **MK937848** | *Sphaerospora ranae* | EF211975 |
| ***Chloromyxum* sp. ex *Bathyraja albomaculata*** | **MK937842** | *Sphaerospora sparidarum* | JX286620 |
| ***Chloromyxum* sp. ex *Bathyraja brachyurops*** | **MK937843** | *Sphaerospora sparis* | JX286624 |
| ***Chloromyxum* sp. ex *Bathyraja macloviana*** | **MK937844** | *Sphaerospora* sp. ex *Ptychadena anchietae* | JX286622 |
| ***Chloromyxum* sp. 3 ex *Carcharhinus limbatus*** | **MK937841** | ***Sphaerospora* sp. 1 ex *Squalus acanthias*** | **MK937853** |
| ***Chloromyxum* sp. ex *Psammobatis rudis*** | **MK937845** | ***Sphaerospora* sp. 2 ex *Mustelus schmitti*** | **MK937854** |
| ***Chloromyxum* sp. ex *Rostroraja eglanteria*** | **MK937846** | ***Sphaerospora* sp*.* 3 ex *Rhizoprionoton terraenovae*** | **MK937855** |
| ***Chloromyxum* sp. ex *Pseudobatos horkelii*** | **MK937847** | *Sphaerospora truttae* | AM410773 |
| ***Chloromyxum* sp. ex *Squatina guggenheim*** | **MK937849** | *Tetracapsuloides bryosalmonae* | KF731712 |
| ***Chloromyxum* sp. ex *Dipturus brevicaudatus*** | **MK937850** | *Zschokkella auratis* | KC849425 |
| ***Chloromyxum squalii*** | **JN130381** | *Zschokkella lophii* | DQ301509 |
| *Chloromyxum thymalli* | JX131381 | *Zschokkella nova* | DQ377690 |
| *Chloromyxum trijugum* | AY954689 |  |  |
| *Chloromyxum truttae* | AJ581916 |  |  |
| ***Chloromyxum zearaji*** | **MG652632** |  |  |
| *Kudoa amamiensis* | AY152748 |  |  |
| ***Kudoa carcharhini*** | **GU324972** |  |  |
| ***Kudoa hemiscylli*** | **GU324958** |  |  |
| *Kudoa inornata* | FJ790311 |  |  |
| *Kudoa iwatai* | AB553294 |  |  |
| *Kudoa musculoliquefaciens* | LC097083 |  |  |
| *Kudoao gawai* | AB636470 |  |  |
| *Kudoa paniformis* | AF034640 |  |  |
| *Kudoa permulticapsula* | AY078429 |  |  |

**Table S3:** Outcome of co-phylogenetic analyses using CoRe-PA.

| Solution | Result | Quality | Total cost | Cospeciations | Sortings | Duplications | Host switches |
| --- | --- | --- | --- | --- | --- | --- | --- |
| **Best ML tree:** | | | | | | | |
| #1 | true | 0.000000000012 | 7.990 | 10 | 39 | 8 | 4 |
| #2 | true | 0.006064085018 | 0.028* | 9 | 68 | 13 | 0 |
| #3 | true | 0.015376294595 | 7.462 | 10 | 45 | 9 | 3 |
| #4 | true | 0.045292864090 | 8.044 | 10 | 35 | 7 | 5 |
| #5 | true | 0.054074461805 | 7.584 | 9 | 53 | 11 | 2 |
| #6 | true | 0.064897524052 | 7.047 | 10 | 60 | 11 | 1 |
| **ML tree with polytomies where bootstrap support <50:** | | | | | | | |
| #1 | true | 0.021389607078 | 8.596 | 8 | 47 | 9 | 5 |
| #2 | true | 0.030570791300 | 8.486 | 6 | 47 | 11 | 5 |
| #3 | true | 0.051525876515 | 8.725 | 8 | 52 | 8 | 6 |
| #4 | true | 0.053274779331 | 8.094 | 8 | 59 | 11 | 3 |
| #5 | true | 0.071282835402 | 7.844 | 8 | 66 | 12 | 2 |
| #6 | true | 0.074665625871 | 8.006 | 6 | 59 | 13 | 3 |

*unlikely event


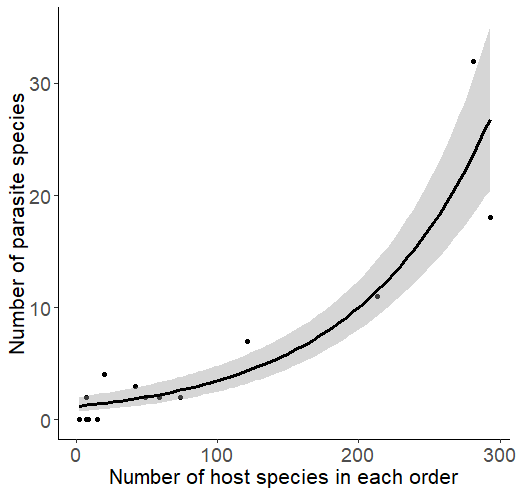


**Figure S1:** Correlation between number of chondrichthyan species and number of myxozoan parasites found in each host order. Statistical analysis was performed using a generalized linear model with Poisson distribution (logarithmic link function) and significance was estimated by the *χ2* criterion (*χ^2^*_1_ = 21.855; *p* < 0.001). Dashed area indicates 95% confidence interval.


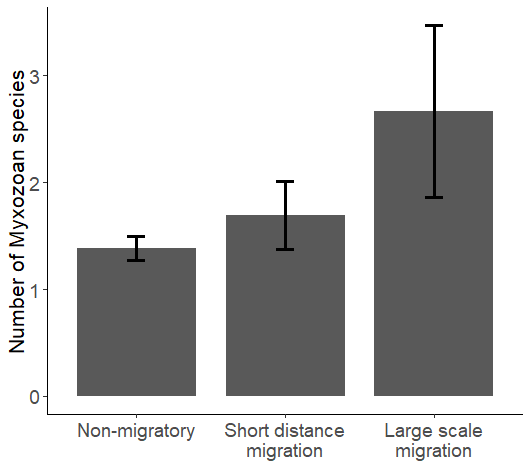


**Figure S2:** Number of myxozoan species in relation to the migration status of their hosts (relates to categories stated in Table S1: 1=non migratory or movements up to 500 m, 2=short seasonal/reproductive migration up to 1000 km; 3=large scale migration >1000km). Statistical analysis employed a generalized linear models with Poisson distribution (logarithmic link function), significance was estimated by the χ2 criterion (χ2=4.36; p = 0.11). Error bars indicate standard error of the mean.


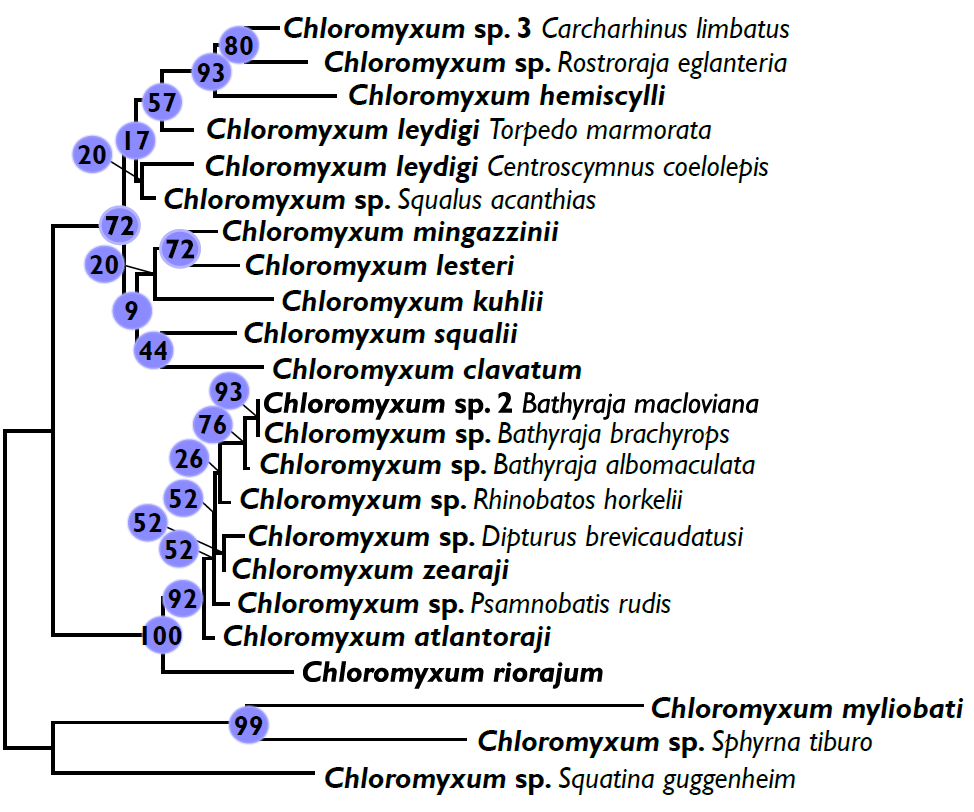


**Figure S3:** Fully resolved ML phylogenetic tree of *Chloromyxum* spp. SSU rDNA sequences. Numbers at nodes indicate bootstrap support.

**
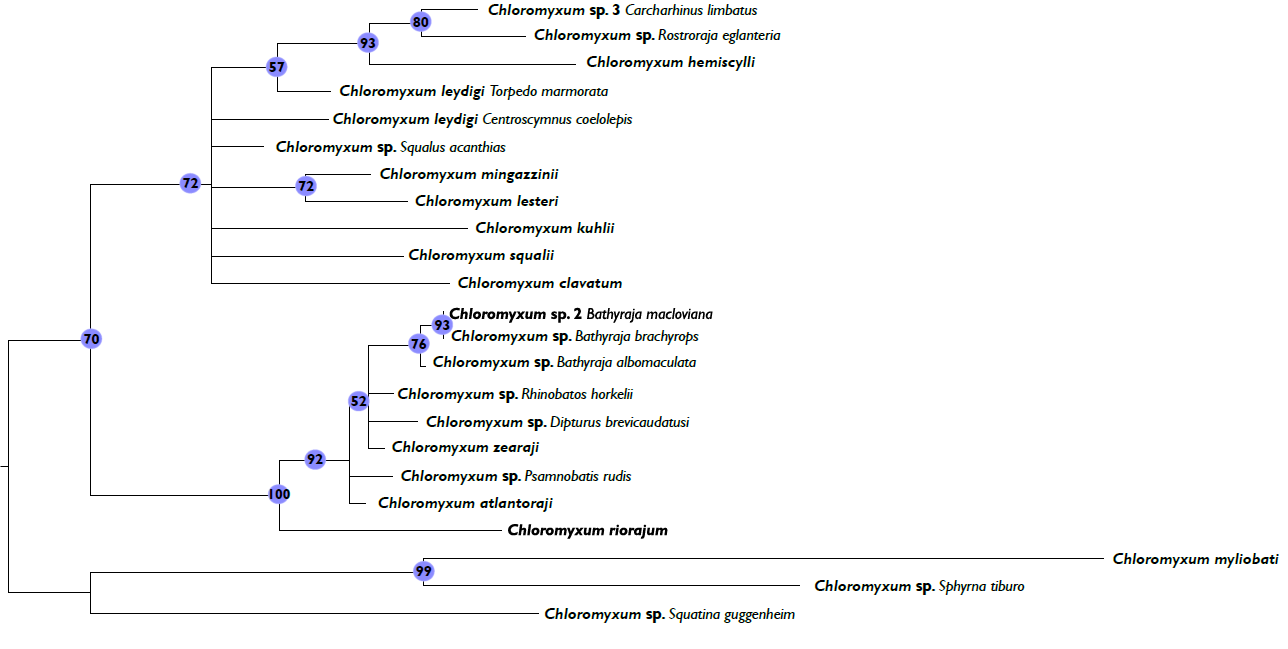
**

**Figure S4:** Partially resolved ML phylogenetic tree of *Chloromyxum* spp. SSU rDNA sequences, with branches showing bootstrap support <50% collapsed. Numbers at nodes indicate bootstrap support.
